# Does the Level of Temporal Demand Affect Activation of the Mental Timeline?

**DOI:** 10.5334/joc.448

**Published:** 2025-05-28

**Authors:** Katharina Kühne, Alex Miklashevsky, Anastasia Malyshevskaya

**Affiliations:** 1 Potsdam Embodied Cognition Group, University of Potsdam, Karl-Liebknecht-Strasse 24–25, House 14, 14476 Potsdam-Golm, Germany; 2 Museum für Naturkunde Berlin, Leibniz-Institut für Evolutions- und Biodiversitätsforschung, Berlin, Germany; 3 Brain Language Laboratory, Department of Philosophy and Humanities, Freie Universität Berlin, Berlin, Germany; 4 Cognitive Health and Intelligence Center, Institute for Cognitive Neuroscience, HSE University, Krivokolenniy Pereulok 3, 101000, Moscow, Russian Federation

**Keywords:** temporal concepts, mental time line, space-time congruency effect, embodied cognition

## Abstract

The space-time congruency effect indicates faster processing of past-/future-related words with the left/right response key, suggesting the presence of the horizontal Mental Time Line (MTL). Typically, this effect is observed in the tasks with high temporal demand (i.e., past versus future categorization), but not in those with the low relevance of the time dimension (i.e., sensicality judgments). However, it remains unclear whether intermediate levels of temporal demand are sufficient to activate the MTL. To address this, we conducted three experiments in which participants categorized the same set of temporal words based on their relation to living entities (Experiment 1), space (Experiment 2), and general time (Experiment 3). In individual analyses of the experiments, the space-time congruency effect was absent in Experiment 1. In Experiment 2, the effect emerged in reaction times but not in accuracy. In Experiment 3, it was observed in both measures. Subsequent comparisons across experiments suggested reliable differences between Experiments 2 and 3 in reaction times and between Experiment 3 and the other two experiments in accuracy. Our results provide evidence that MTL activation depends on the level of temporal demand required by the task. The findings support the notion that mental representations are context-sensitive rather than fixed.

## 1. Introduction

Time is not directly accessible to sensory perception: It cannot be touched, seen, heard, smelled, or tasted. Yet, humans have developed robust ways of understanding time. One prominent explanation in cognitive science is that humans conceptualize time by relying on a more directly perceivable domain – space. Indeed, space and time are processed in overlapping brain regions, specifically within parietal and frontal areas (Bueti & Walsh, 2009; Cona et al., 2021; Walsh, 2003). The association between these domains is further supported by clinical evidence. Patients with hemineglect – a condition characterized by the inability to attend to stimuli on one side of space – often display similar impairments in their ability to process time (Huberle & Brugger, 2018). The link between time and space is also evident in external human behavior. For instance, representatives of various cultures use spontaneous gestures to map past or future events onto specific spatial dimensions when discussing time (Casasanto, & Jasmin, 2012; Núñez et al., 2012; Walker & Cooperrider, 2016). Additionally, time is frequently represented in spatial formats, such as calendars, timelines, or circles. Finally, temporal expressions in many languages incorporate spatial semantics, such as “looking forward to the future”, “putting the past behind us”, or describing events as being “far apart” in time (Lakoff, 1993).

Importantly, recent research has shown that the processing of time-related words is accompanied by lateral spatial biases (e.g., Chrabaszcz et al., 2024). One well-investigated effect related to this phenomenon is the space-time congruency effect: Words referring to the past (e.g., “yesterday”) are responded to faster with the left key, while words referring to the future (e.g., “tomorrow”) are responded to faster with the right key (see von Sobbe et al., 2019, for meta-analysis). This effect might be explained by attentional shifts akin to the classical Posner cueing paradigm (Posner, 1980): As an analogue to spatial cues, time-related words bias participants’ spatial attention facilitating faster responses in congruent conditions in comparison to incongruent ones. The space-time congruency effect suggests that the mental representation of temporal concepts unfolds from the past to the future along a horizontal Mental Time Line (MTL; Bonato et al., 2012; Boroditsky, 2001; Clark, 1973; Fuhrman et al., 2011; Fuhrman & Boroditsky, 2010; Moore, 2006; Santiago et al., 2010; see for review Bender & Beller, 2014; Grasso, Ziegler, Coull, et al., 2022b; Núñez & Cooperrider, 2013; Núñez & Sweetser, 2006; Spatola et al., 2018). This horizontal spatial-temporal mapping has been observed across various types of temporal concepts, including deictic time-related words (e.g., “yesterday”, “tomorrow”; Malyshevskaya, Miklashevsky, et al., 2024; Woodin & Winter, 2018), verbs in past and future tense (e.g., “went”, “will go”; Chrabaszcz et al., 2024; Grasso, Ziegler, Coull, et al., 2022a) and words representing time units (e.g., “Monday”, “January”; Gevers et al., 2003, 2004; Malyshevskaya et al., 2022; Malyshevskaya, Miklashevsky, et al., 2024). Moreover, MTL activation has been demonstrated in tasks using visual and auditorial modalities (Kong & You, 2012; Lakens et al., 2011; Ouellet et al., 2010) and both in language production (Núñez & Cooperrider, 2013) and comprehension (Grasso, Ziegler, Coull, et al., 2022b).

While a substantial body of research supports the widespread existence of the space-time congruency effect, its underlying mechanisms remain a topic of ongoing debate (e.g., Maienborn et al., 2015). Recent studies have specifically focused on identifying the conditions under which signatures of MTL activation can be detected (Santiago et al., 2011; Scheifele et al., 2018; Sell & Kaschak, 2011). Notably, the space-time congruency effect has been most commonly observed in tasks with high temporal demand, such as when participants categorize words based on their temporal dimension (i.e., past versus future; Bottini et al., 2015; De La Vega et al., 2016; Ding et al., 2015; Eikmeier et al., 2015; Flumini & Santiago, 2013; Fuhrman et al., 2011; Fuhrman & Boroditsky, 2007; Maienborn et al., 2015; Ouellet et al., 2010; Ulrich et al., 2012; Walker et al., 2014). In contrast, no effect has been reported by the majority of studies using less temporally demanding tasks, such as those involving sensicality judgments (determining whether a sentence containing a temporal referent is meaningful; Maienborn et al., 2015; Scheifele et al., 2018; Sell & Kaschak, 2011; Ulrich et al., 2012; Ulrich & Maienborn, 2010). In the recent meta-analysis, von Sobbe and colleagues (2019) estimated average effect sizes in tasks with low and high temporal demand and concluded that in the former, the effect does not differ significantly from zero.

These and similar findings have led some researchers to conclude that MTL activation is not functionally involved in surface levels of conceptual processing (Maienborn et al., 2015; Santiago et al., 2011, 2012; Torralbo et al., 2006; Ulrich & Maienborn, 2010). At the same time, theories of cognition suggest that mental representations are not fixed but sensitive to context and environment (Barsalou, 1982, 2020; Fischer, 2012; Kaup et al., 2024; Kiefer & Pulvermüller, 2012; Louwerse & Jeuniaux, 2008; Myachykov et al., 2014). Specifically, some researchers suggested that deeper conceptual processing might lead to stronger involvement of mental representations resulting in stronger sensorimotor simulation or attentional shifts (Ibáñez et al., 2022; Myachykov et al., 2014; Pulvermüller, 2018; Shaki & Fischer, 2023). Regarding time-related words, not only do they denote a specific temporal dimension (e.g., past, present, or future) but also include other semantic aspects activated depending on the task contextual demands. Thus, time-related words can be processed at different levels of temporal demand, from the deepest (temporal dimension) to more surface ones. Following this view, one could hypothesize that attentional biases accompanying the activation of temporal concepts should be observed in tasks with intermediate levels of temporal demand. In particular, the strength of attentional biases should increase with increasing temporal demands (that is, more pronounced salience of the word temporal dimension). However, the existing MTL research (and consequently the meta-analysis by von Sobbe et al., 2019) is limited by the narrow variability of experimental tasks involving semantic processing. Specifically, the meta-analysis primarily included tasks focused on either past versus future categorization or sentence sensicality judgment. The lack of intermediate levels of temporal demands limits the range to these two extremes (Maienborn et al., 2015).

The idea that attentional biases should vary depending on the level of temporal demands is partially supported by recent experimental results. One of them is the research by Malyshevskaya and colleagues (2024), which demonstrated the horizontal MTL activation while the word temporal dimension was kept subtle to the task. The authors used words related to hours of the day (e.g., “8 a.m.”), days of the week (e.g., “Monday”), and months of the year (e.g., “November”). A line bisection task was implemented: Participants clicked on the dot to start the presentation of a temporal word and then indicated the center of horizontal lines. Lateral biases in participants’ manual responses caused by processing the words were assessed. The authors found that participants deviated more to the left when processing left-biasing time units and more to the right when processing right-biasing ones.

Importantly, Malyshevskaya and colleagues (2024) did not focus on the word temporal dimension (i.e., relatedness to past vs. future) in their task. Instead, they asked participants to process temporal concepts based on a broader reference – the class of the time unit. Nevertheless, this level of temporal demand proved sufficient to activate the horizontal MTL. However, one could argue that time units represent ordinal sequences, and their nature is different from that of past- and future-related words, such as “yesterday” or “tomorrow” (Abrahamse et al., 2017). Thus, to transfer the idea from Malyshevskaya and colleagues (2024) to deictic time-related words, one could use their broader reference to the concept of time *itself*. Indeed, deciding whether the word refers to time or not might represent an intermediate level of temporal demand: Due to the high salience of time semantics, it closely approximates the classic past versus future categorization while keeping the word temporal dimension irrelevant to the task. In the present study, we use this novel approach – decision regarding the word time-relatedness – as one of the intermediate-level tasks selected to investigate the level of temporal demand sufficient for MTL activation.

Further evidence suggesting varying levels of processing temporal concepts comes from studies investigating the bidirectionality of the association between space and time. For instance, Anelli and colleagues (2016) manipulated participants’ spatial attention using prismatic adaptation. Participants adapted to a visual distortion induced by prism glasses, which laterally shifted the visual field (either leftward or rightward) by refracting light rays. During this manipulation, participants were asked to mentally project themselves into the past, present, or future and categorize events based on the temporal dimension (past versus future). The study revealed that a leftward shift in spatial attention facilitated responses to past-related events, whereas a rightward shift facilitated responses to future-related events. Such modulation of temporal information processing through lateral spatial biases has been demonstrated in numerous other studies (e.g., Anelli & Frassinetti, 2019; Casadio et al., 2024). Importantly, *spatial language* modulates processing of temporal concepts. For example, Akbuğa and Goksun (2024) presented participants with temporal expressions that either lacked spatial words or contained metaphors referencing sagittal or non-sagittal space. They found that words related to sagittal space elicited sagittal representations of time, whereas temporal expressions without spatial references or involving non-sagittal metaphors led to activation of the horizontal spatial-temporal mapping.

Based on the findings suggesting the bidirectionality of the association between space and time, it can be hypothesized that focusing on the spatial semantic aspect of a word may influence the activation of its temporal meaning to some extent. Indeed, temporal and spatial semantics are often interconnected, as demonstrated by their frequent co-occurrence in temporal metaphors such as “moving the meeting forward”, “the weekend is behind us”, or “going back to childhood” (Lakoff & Johnson, 1999). Consequently, another task that may engage an intermediate level of temporal demand involves identifying the spatial semantic aspect of a word – that is, determining whether the word is related to space or not. Here, we hypothesize that focusing on a spatial semantic aspect might lead to greater temporal demand compared to less relevant aspects. To the best of our knowledge, no MTL studies to date have specifically utilized this task in relation to temporal concepts. In the present study, we use the space-relatedness task as a novel way to assess MTL activation while ensuring that the word temporal dimension remains subtle.

Overall, the level of temporal demand required to activate spatial biases accompanying understanding of time-related words remains a subject of ongoing debate. To address this issue, we examined different levels of temporal demand under a unified experimental paradigm while avoiding direct categorization of the word temporal dimension (past versus future). Across three experiments, we used the same set of past- and future-related words, and participants categorized these words by using left and right response keys. Reaction times and accuracy were measured as dependent variables, while response side (left versus right) and words’ time position (from past to future, see Methods for details) were included as independent variables in all experiments.

At the same time, each experiment employed a different word categorization task, thereby engaging distinct levels of temporal demand. We progressed from the lower to higher levels of temporal demand while keeping the word temporal dimension subtle to the task. As a baseline, Experiment 1 used a task requiring the least temporal demand. Note that the sensicality judgment task extensively used in previous studies (Maienborn et al., 2015; Scheifele et al., 2018; Sell & Kaschak, 2011; Ulrich et al., 2012; Ulrich & Maienborn, 2010) is only applicable at the sentence level. Since we used single words, we were confronted with the need to find an alternative semantic task. Previous research demonstrated that the categorization of words as living or non-living entities involves a core semantic dimension activated largely automatically (Bonin et al., 2019). At the same time, the word animacy task is improbable to elicit temporal demands. Therefore, we selected the word animacy task for Experiment 1. In Experiment 2, we asked participants to categorize words according to their space-relatedness. Since spatial words play an important role in the conceptualization of time, this task presumably represents an intermediate level of temporal demand in our study. Finally, in Experiment 3, we employed a time-relatedness task. Due to the focusing on the time itself, this task has a higher temporal demand than the space-relatedness task, yet keeps the temporal dimension (past versus future) subtle.

Based on the aforementioned arguments, we proposed the following hypotheses:

1. Because no space-time congruency effect has been observed in the majority of studies avoiding temporal semantic aspect of the word (von Sobbe et al., 2019), we expected that the space-time congruency effect *should not be observed* in the word animacy task (Experiment 1).
2. Because temporal and spatial semantics are often interconnected (Lakoff & Johnson, 1999) and spatial words might modulate the retrieval of specific spatial-temporal mapping (Akbuğa & Göksun, 2024), we expected that the space-time congruency effect *should be observed* in the space-relatedness task (Experiment 2).
3. Because previous studies reported the space-time congruency effect in tasks focusing on a broader temporal reference than the word temporal dimension (Malyshevskaya, Miklashevsky, et al., 2024), we expected that the space-time congruency effect *would be observed* in our time-relatedness task (Experiment 3).

## 2. Experiment 1: Animacy task

### 2.1. Methods

#### 2.1.1. Sample size calculation

According to the meta-analysis by von Sobbe and colleagues (2019), the space-time congruency effect should be of medium size when past versus future categorization is required (*d* = 0.46). The authors recommend using this mean effect size as an estimate for future experiments employing a within-participant ANOVA design and suggest collecting a minimum of 41 participants to detect the space-time congruency effect (*α* = .05, *power* = .90). Since mixed-effects models used in the present study are even more powerful than standard separate analyses by participants and by items (Brysbaert & Stevens, 2018), using mixed-effects modeling additionally ensured our ability to detect effects of interest if present. Expecting no effect in the task avoiding temporal semantic aspect of the word, we sought to find at least moderate evidence for the null hypothesis. For this, at least 60 participants would be needed in follow-up sensitivity Bayesian analyses (Brysbaert, 2019). Considering possible drop-outs (around 10% based on our previous experience with online studies), we aimed at a minimal sample size of 66 participants.

#### 2.1.2. Stimuli

As some recent studies suggested that MTL activation occurs in a continuous manner in tasks avoiding past versus future categorization (Beracci et al., 2022), we used time-related stimuli representing a temporal spectrum (ranging from distant past, through recent past and near future, to distant future) and incorporated this relative time position as a continuous variable in our analysis. We used 20 adverbs related to future (for example, “bald”, *soon*), and 20 adverbs related to past (for example, “gestern”, *yesterday*). These stimuli were selected based on the pretest with native German speakers (*N* = 40; 36 females; *Mean* age = 24 years; all native speakers of German) who evaluated word past- vs. future-relatedness on a scale from 1 (“related to very distant past”) through 5 (“related to the present moment”) to 9 (“related to very distant future”). The values for past-related words ranged from 1.5 to 4.05; the values for future-related words ranged from 5.17 to 8.4. The stimuli selection procedure also controlled for word length (in letters) and frequency (lgSUBTLEX, see Brysbaert et al., 2011). The pretest was conducted by Dimitrov (Dimitrov, 2021; see that thesis for further details of the pretest study). Descriptive statistics of the time-related words are presented in Table 1. See Appendix A for the complete list of time-related words and their characteristics. All stimuli were displayed in capital letters in the center of the screen in Sans-serif font, 36 pt, black on a white background.

**Table 1:** Descriptive statistics of time-related stimuli.


VARIABLE	PAST-RELATED WORDS	FUTURE-RELATED WORDS	COMPARISON (*T*-TEST)

Time position	3.065 (0.937)	6.521 (0.721)	*t*(38) = 12.741, *p* < .001

Length	7.150 (1.526)	7.750 (1.997)	*t*(38) = 1.041, *p* = .305

Frequency	2.321 (0.984)	2.316 (1.133)	*t*(38) = –0.013, *p* = .990


*Note*. Time position could range from 1 (“related to very distant past”) through 5 (“related to the present moment”) to 9 (“related to very distant future”) and was measured in units on a Likert scale; see main text for details. Numbers in cells indicate mean values, and numbers in parentheses indicate SDs. Length was measured in letters. Frequency was measured in ipm (items per million).

As time-unrelated stimuli, we selected 80 animate nouns (for example, “Fuchs”, *fox*), 20 inanimate object names (for example, “Bürste”, *brush*) and 20 abstract adverbs not related to time (for example, “ebenso”, *likewise*) to disguise the purpose of the experiment. Animate and inanimate words (with the latter category including time-related adverbs, time-unrelated abstract adverbs, and inanimate object names) did not differ significantly from each other in length (*t*(158) = –1.925, *p* = .056) or frequency (*t*(158) = –1.588, *p* = .114).

Time-related and non-related words did not differ significantly in frequency (lgSUBTLEX, see Brysbaert et al., 2011) and length. See Table 2 for descriptive statistics of both types of stimuli.

**Table 2:** Descriptive statistics for time-related vs. non-related words in Experiment 1.


	TIME-RELATED WORDS		TIME NON-RELATED WORDS		COMPARISON (T-TEST)	
		
	*MEAN*	*SD*	*MEAN*	*SD*	*T*	*P*

Length	7.450	1.825	6.783	2.235	–1.705	.090

Frequency	2.319	1.075	2.049	0.701	–1.827	.070


*Note*. In Experiment 1, the non-time category included 120 stimuli, while the time category had 40 stimuli (20 past- and 20 future-related; see Table 1).

#### 2.1.3. Design and procedure

The experiment was programmed and run using the online Gorilla Experiment Builder service (www.gorilla.sc, Anwyl-Irvine et al., 2020). Participants were presented with the word (until response or timed out after 1,500 ms). After 1,500 ms, participants could not respond. The word presentation was followed by an inter-trial interval (1,000 ms) during which ####### appeared on the screen. Participants first had a short practice part (16 trials) in which they received feedback. If their response was correct, a green tick appeared on the screen; a red cross on the screen followed incorrect responses. No feedback was provided in experimental trials. The stimuli were delivered in a randomized order. Accuracy and reaction time (RT) were measured. After the practice, participants continued with the main task. Keys Q and P (i.e., located on the left and right, respectively) were used for responses. The order of the blocks (response mapping) was counterbalanced within participants. Experiment 1 consisted of 320 trials. Each word was presented twice per experiment (once per block). After every 40 trials, participants were suggested to take a break. After finishing the experiment, participants were asked to fill in a demographic questionnaire, including questions about their age, gender, and native language, as well as a short version of the Edinburgh handedness inventory (Veale, 2014). The entire testing session lasted around 30 minutes.

The study was designed and conducted following the guidelines laid down in the Declaration of Helsinki. All participants submitted their informed consent at the beginning of the experiment by clicking on the corresponding checkbox. Participants were recruited among students of the University of Potsdam by using the SONA Participant Pool (https://www.uni-potsdam.de/en/sona-kogwis/index) and reimbursed with course credits.

#### 2.1.4. Task

Participants used the Q and P keys to respond, with one key for animate words and the other for inanimate words, depending on the block instruction. Each participant completed both response mappings, each assigned to a different block. The block order was counterbalanced between participants.

#### 2.1.5. Participants

Initially, 117 university students took part in the online experiment. Data preparation and processing in this and all the following analyses were done using R (R Core Team, 2020). All processing scripts are available online (see Data Accessibility Statement). We excluded from the main sample non-native speakers of German, non-right-handed participants (i.e., those having EHI score lower than +50), those who reported that they did not perform the task seriously, those who reported having conducted the study in a very loud environment and/or having technical difficulties during the experiment, and those who did not fill out the questionnaires at the end of the session (*N* = 8 in all these categories). Next, we removed one participant with a mean accuracy of ≤75% in the task. The data of the remaining 108 participants (94 female, 13 male, and 1 non-binary; *Mean* age = 23 years; *SD* = 5 years; *Mean* EHI score = 95, *SD* = 11) were submitted for further analyses.

The criteria for excluding outlier data, including both participants and individual trials, were established during the data analysis process based on the inspection of error rates (which depend on task difficulty and are difficult to estimate in advance for novel tasks like ours), the distribution of accuracy across the entire sample in each experiment, and data-quality checks. For example, we compared accuracy and mean RTs between participants who reported severe technical issues during the experiment and those who did not, identified participants who completed the experiment but did not fill out the questionnaire, or examined the number of non-right-handers and determined that it was small enough to exclude them to maintain a homogeneous sample without losing too much data. These criteria were defined for each experiment, then aligned across all experiments and consistently applied in each one.

### 2.2. Analysis and results

#### 2.2.1. Reaction times

The average accuracy of the remaining participants was 95% (*SD* = 4%), and the average RT was 615 ms (*SD* = 72 ms). We excluded anticipation responses, i.e., responses faster than 200 ms (< 1% of the data). There were no responses longer than 1,500 ms. We also excluded error trials (5% of the data) and RTs lying outside three *SD*s from participants’ individual *Mean* RTs (2% of the data). We discarded all time-unrelated words. The remaining 8,228 trials with time-related stimuli were submitted for further analysis.

All continuous variables were mean-centered, and sum-coded contrasts (–0.5 and 0.5) were assigned to the categorical variable Response Side (Barr et al., 2013). The data were submitted to a linear mixed model analysis using the R package *lme4* (Bates et al., 2015). In the initial model, we included Time Position, Response Side, and the key interaction between Time Position and Response Side as fixed factors; additionally, we included Trial Number, Length, and Frequency as covariates. The models with random slopes for the interaction between Time Position and Response Side (by participant) or the main effect of Response Side (by item) did not converge; thus, we only kept random intercepts for participants and items. We performed a backward elimination using the *drop1* function to identify the best-fit model; effects that did not improve model fit (*p* > .100) were successively eliminated. The main variables of interest (Time Position and Response Side) and their interaction are reported regardless of significance.

**Figure 1:**
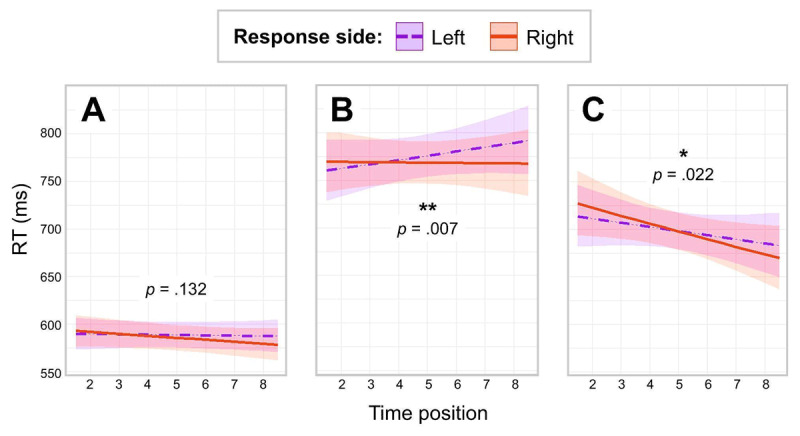
Predicted RTs as a function of Response Side and Time Position in three experiments. *Note*. Panel A: Results of Experiment 1 (word animacy task). Panel B: Results of Experiment 2 (space-relatedness task). Panel C: Results of Experiment 3 (time-relatedness task).

The detailed results from the best-fit model are presented in Table B1 in Appendix B. Neither Time Position (*b* = –0.0020, *t* = –0.9026, *p* = .367) nor Response Side (*b* = –0.0045, *t* = –1.2060, *p* = .228) were significant. The interaction between the two factors was not significant (*b* = –0.0030, *t* = –1.5077, *p* = .132). In other words, there was no evidence of MTL activation in RTs in this experiment. See Figure 1A for results.

#### 2.2.2. Accuracy

To examine participants’ accuracy and also to exclude the possibility of a speed-accuracy trade-off (Heitz, 2014) we conducted an accuracy analysis. The same approach was used as for RTs except that in this analysis, we did not exclude error trials. Anticipation responses were excluded, i.e., responses faster than 200 ms (< 1% of the data). We also excluded RTs lying outside three *SD*s from participants’ individual *Mean* RTs (2% of the data). All time-unrelated stimuli were discarded. The remaining 8,751 trials with time-related words were submitted into further analysis. As before, continuous variables were mean-centered, and sum-coded contrasts (–0.5 and 0.5) were assigned to the categorical variable Response Side. We submitted the data to a generalized linear mixed-effects model (function *glmer*) with random intercepts for participants and items. This time, we did not use backward elimination. Instead, we employed the same list of variables that were already revealed in the final model for RT analysis described just above to keep the results for accuracy comparable with those for RTs.

**Figure 2:**
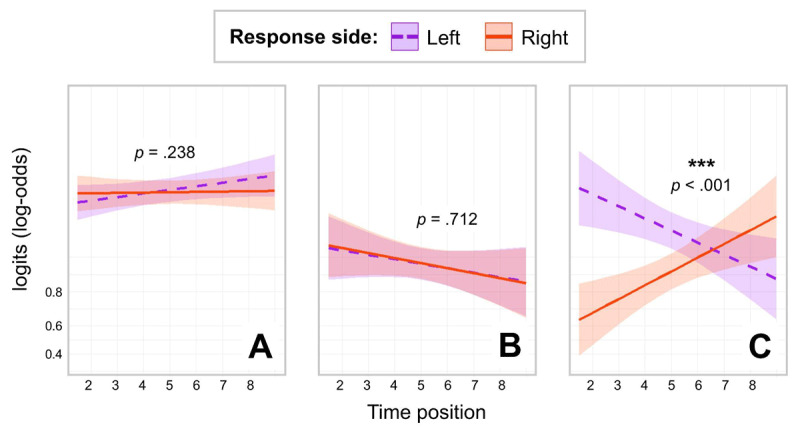
Results for the analysis of Accuracy as a function of Response Side and Time Position in three experiments. *Note*. Panel A: Results of Experiment 1 (word animacy task). Panel B: Results of Experiment 2 (space-relatedness task). Panel C: Results of Experiment 3 (time-relatedness task). The Y-axis represents logits (log-odds) to display the linear trend. For back-transformed accuracy values, see Figure D1 in Appendix.

The model output for accuracy is presented in Table B2 in Appendix B. Neither Time Position (*OR* = 1.0579, *z* = 1.3222, *p* = .186) nor Response Side (*OR* = 0.9348, *z* = –0.4403, *p* = .660) were significant. The interaction between the two factors was also not significant (*OR* = 0.9117, *z* = –1.1797, *p* = .238). See Figure 2A for results. Interested readers can also consult Appendix C for descriptive statistics of actual performance data, i.e., accuracy data and reaction time data without covariates.

### 2.3. Discussion

As expected, Experiment 1 demonstrated no significant space-time congruency effect. In the following experiment, we examined MTL activation in the space-relatedness task. This task presumably represented an intermediate level of temporal demand since spatial words play an important role in the conceptualization of time.

## 3. Experiment 2: Space-relatedness task

### 3.1. Methods

#### 3.1.1. Sample size calculation

Sample size calculation followed the same logic as in Experiment 1. A minimum of 41 participants was required to detect the space-time congruency effect (*α* = .05, *power* = .90).

#### 3.1.2. Stimuli

For Experiment 2, we used the same set of 20 German adverbs related to future (for example, “morgen”, *tomorrow*) and 20 German adverbs related to past (for example, “neulich”, *recently*) as in Experiment 1 (see Section 2.1.2). Additionally, we selected 80 German adverbs related to space (for example, “bergab”, *downhill*) and 40 German adverbs belonging to neither time or space categories (for example, “leider”, *unfortunately*). (See Table 3 for further stimuli details.) The spatial relatedness of the stimuli was assessed in a separate study with native German speakers who chose one of the options: “The word has a spatial meaning” (+1), “I am not sure” (0), or “The word has no spatial meaning” (–1) for each of the words. Therefore, we were able to calculate spatial relatedness for each word as a value from +1 (strongly spatial) to –1 (absolutely not spatial). Spatial words were significantly more related to space (*Mean* = 0.93, *SD* = 0.09) than non-spatial words (*Mean* = –0.76, *SD* = 0.33; *t*(158) = 43.7, *p* < .001). Past-related (*Mean* = –0.60, *SD* = 0.38) and future-related words (*Mean* = –0.55, *SD* = 0.40) did not differ significantly from each other in terms of spatial relatedness (*t*(38) = –0.345, *p* = .732). The space-related words were matched with the other group in length and frequency (lgSUBTLEX, Brysbaert et al., 2011).

**Table 3:** Descriptive statistics for time-related vs. non-related words in Experiment 2.


	TIME-RELATED WORDS		TIME NON-RELATED WORDS		COMPARISON (T-TEST)	
		
	*MEAN*	*SD*	*MEAN*	*SD*	*T*	*P*

Length	7.450	1.825	7.275	1.763	–0.539	.591

Frequency	2.319	1.075	2.373	1.130	0.265	.791


*Note*. In Experiment 2, the non-time category included 120 stimuli, while the time category had 40 stimuli (20 past- and 20 future-related; see Table 1).

#### 3.1.3. Design and procedure

The design and procedure were identical to Experiment 1 apart from the stimuli material and the task. Experiment 2 consisted of 320 trials. As before, each word was presented twice per experiment (one time per block).

#### 3.1.4. Task

Participants used the Q and P keys to respond, pressing one for space-related words and the other for non-space-related words, depending on the block instruction. Each participant completed both response mappings, with one mapping assigned to each block. The block order was counterbalanced between participants.

#### 3.1.5. Participants

Initially, 116 university students took part in the online experiment. We used the same exclusion criteria as in Experiment 1 (*N* = 8 in all excluded categories). We also removed three participants with a mean accuracy of ≤75% in the task. The data of the remaining 105 participants (86 female, 19 male; *Mean* age = 23 years; *SD* = 6 years; *Mean* EHI score = 95, *SD* = 12) were submitted for further analyses.

### 3.2. Analysis and results

#### 3.2.1. Reaction times

The average accuracy of the remaining participants was 91% (*SD* = 3%), and the average RT was 754 ms (*SD* = 84 ms). As before, we excluded anticipation responses, i.e., responses faster than 200 ms (< 1% of the data). There were no responses longer than 1,500 ms. We also excluded error trials (9% of the data) and RTs lying outside three SDs from participants’ individual Mean RTs (1% of the data). We discarded all time-unrelated words. The remaining 6,767 trials with time-related stimuli were submitted for further analysis.

We used the same analytical approach as in Experiment 1. The detailed results from the best-fit model are presented in Table B3 in Appendix B. There was no significant main effect of Time Position (*b* = 0.0027, *t* = 0.5850, *p* = .559). There was a marginally significant main effect of Response Side (*b* = –0.0076, *t* = –1.6574, *p* = .097), with a tendency to respond slower with the left hand. Importantly, the interaction between the two factors was significant (*b* = –0.0062, *t* = –2.7039, *p* = .007). To examine this interaction, we releveled the dataset separately for each of the two levels of Response Side. There was a non-significant positive main effect of Time Position in left-hand responses (*b* = 0.0058, *t* = 1.2077, *p* = .227), and a non-significant negative effect of Time Position in right-hand responses (*b* = –0.0003, *t* = –0.0707, *p* = .944). It is important to note that when releveling is employed, the slopes are compared to zero. While the slopes may differ substantially from one another, particularly when they trend in opposite directions, they may still not exhibit significant deviations from zero in isolated comparisons, as demonstrated in this instance. Therefore, one can still conclude that participants responded significantly (see the interaction) differently to time-related words depending on word semantic and response key, with faster responses to past-related words with their left hand and to future-related words with their right hand, as the second hypothesis predicts. See Figure 1B for results.

#### 3.2.2. Accuracy

As in Experiment 1, we conducted an accuracy analysis to examine participants’ accuracy and also to exclude the possibility of a speed-accuracy trade-off. The same approach was used as for RTs except that in this analysis, we did not exclude error trials. The 8,090 trials with time-related stimuli were submitted into a generalized linear mixed-effects model (function *glmer*) with random intercepts for participants and items. Instead of using a backward elimination, we employed the same list of variables that were already revealed in the final model for RT analysis described just above to keep the results for accuracy comparable with those for RTs. The model output for accuracy is presented in Table B4 in Appendix B.

Neither Time Position (*OR* = 0.8731, *z* = –1.2127, *p* = .225) nor Response Side (*OR* = 1.0260, *z* = 0.3519, *p* = .725) were significant. The interaction between the two factors was not significant (*OR* = 0.9841, *z* = –0.3697, *p* = .712). See Figure 2B for results. The reader can also consult Appendix C for descriptive statistics of actual performance data, i.e., accuracy data and reaction time data without covariates.

In other words, while there was a significant interaction between Time Position and Response Side in RTs, this interaction was not significant in accuracy. It suggests that there was no indication of a speed-accuracy trade-off in Experiment 2.

### 3.3. Discussion

In Experiment 2, we found a reliable space-time congruency effect in RTs: There was a significant interaction between Time Position and Response Side. According to our second hypothesis, participants responded faster to past-related words with the left key and to future-related words with the right key. Note that while participants processed words based on their spatial semantics, time still remained a highly subtle semantic dimension in this experiment. In Experiment 3, we asked participants to evaluate the time-relevance of stimuli. Due to the relation to time itself, this task exhibited the highest level of temporal demand yet kept the word temporal dimension irrelevant to the task performance.

## 4. Experiment 3: Time-relatedness task

### 4.1. Methods

#### 4.1.1. Sample size calculation

Sample size calculation followed the same logic as in Experiments 1 and 2. A minimum of 41 participants was required to detect the space-time congruency effect (*α* = .05, *power* = .90).

#### 4.1.2. Stimuli

For Experiment 3, we used the same 40 German time-related adverbs as in Experiment 1 and 2 (for example, “gestern”, *yesterday*, or “nachher”, *then*). In addition, we used 40 other German adverbs: abstract (for example, “eigentlich”, *actually*), selected from the stimuli pool of Experiment 1 and space-related (for example, “drunten”*, below*), selected from the stimuli pool of Experiment 2. The time-related stimuli were matched with the counter-group in length and frequency (lgSUBTLEX, Brysbaert et al., 2011). (See Table 4.)

**Table 4:** Descriptive statistics for time-related vs. non-related words in Experiment 3.


	TIME-RELATED WORDS		TIME NON-RELATED WORDS		COMPARISON (T-TEST)	
		
	*MEAN*	*SD*	*MEAN*	*SD*	*T*	*P*

Length	7.450	1.825	7.550	1.853	0.243	.809

Frequency	2.319	1.075	2.621	0.954	1.331	.187


*Note*. In Experiment 3, both the non-time and time categories included 40 stimuli, with the latter consisting of 20 past- and 20 future-related stimuli (see Table 1).

#### 4.1.3. Design and procedure

The design and procedure were identical to Experiment 1 and Experiment 2, apart from the stimuli material and the task. Experiment 3 consisted of 160 trials. As before, each word was presented twice per experiment (one time per block).

#### 4.1.4. Task

Participants used the Q and P keys to respond, pressing one for time-related words and the other for time-unrelated words, depending on the block instruction. Each participant completed both response mappings, with one mapping assigned to each block. The block order was counterbalanced between participants.

#### 4.1.5. Participants

Initially, 120 university students took part in the online experiment. We used the same exclusion criteria as in Experiments 1 and 2 (*N* = 6 in all excluded categories). We also removed two participants with a mean accuracy of ≤75% in the task. The data of the remaining 113 participants (87 female, 22 male, 3 non-binary, and 1 with no response; *Mean* age = 24 years; *SD* = 6 years; *Mean* EHI score = 94, *SD* = 13) were submitted to further analyses.

### 4.2. Analysis and results

#### 4.2.1. Reaction times

The average accuracy of the remaining participants was 90% (*SD* = 4%), and the average RT was 739 ms (*SD* = 75 ms). We excluded anticipation responses, i.e., responses faster than 200 ms (< 1% of the data). There were no responses longer than 1,500 ms. We also excluded error trials (10% of the data) and RTs lying outside three *SD*s from participants’ individual *Mean* RTs (1% of the data). All time-unrelated words were discarded. The remaining 7,543 trials with time-related stimuli were submitted for further analysis. We used the same analytical approach as in Experiments 1 and 2.

The detailed results from the best-fit model are presented in Table B5 in Appendix B. There was no significant main effect of either Time Position (*b* = –0.0090, *t* = –1.6101, *p* = .107) or Response Side (*b* = 0.0002, *t* = 0.0522, *p* = .958). Crucially, there was a significant interaction between the two factors (*b* = –0.0054, *t* = –2.2983, *p* = .022). To examine this interaction, we releveled the dataset separately for each of the two levels of Response Side. There was a non-significant negative main effect of Time Position in left-hand responses (*b* = –0.0063, *t* = –1.1091, *p* = .267) and a significant negative main effect of Time Position in right-hand responses (*b* = –0.0117, *t* = –2.0334, *p* = .042). In other words, participants responded significantly faster with the right key to future-related than to past-related stimuli. See Figure 1C for results.

#### 4.2.2. Accuracy

As in Experiments 1 and 2, to examine participants’ accuracy and also to exclude the possibility of a speed-accuracy trade-off we conducted an accuracy analysis. The same approach was used as for RTs except that in this analysis, we did not exclude error trials. The 8,751 trials with time-related stimuli were submitted into a generalized linear mixed-effects model (function *glmer*) with random intercepts for participants and items. Instead of using a backward elimination, we employed the same list of variables that were already revealed in the final model for RT analysis described just above to keep the results for accuracy comparable with those for RTs. The model output for accuracy is presented in Table B6 in Appendix B.

Time Position was not significant (*OR* = 1.1103, *z* = 0.9596, *p* = .337) while Response Side was (*OR* = 0.4620, *z* = –9.7494, *p* < .001), with more accurate responses on the left side. Most importantly, the interaction between the two factors was significant (*OR* = 1.8790, *z* = 15.2637, *p* < .001). To examine this interaction, we releveled the dataset separately for each of the two levels of Response Side. There was a marginally significant negative main effect of Time Position in left-hand responses (*OR* = 0.8100, *z* = –1.8911, *p* = .059) and a significant positive main effect of Time Position in right-hand responses (*OR* = 1.5220, *z* = 3.8007, *p* < .001). In other words, participants responded significantly more accurately with their right hand to future-related than to past-related stimuli, while a reverse tendency was observed in the left hand (though non-significant). See Figure 2C for results. Interested readers can also consult Appendix C for descriptive statistics of actual performance data, i.e., accuracy data and reaction time data without covariates.

The results of the accuracy analysis suggest that there was no speed-accuracy trade-off: Participants were both significantly faster and more accurate when responding to future-related stimuli with the right key. While participants tended (*p* = .059) to respond more accurately to past-related stimuli with the left key, we did not find a significant effect of Time Position in left-hand RTs, which also provides no evidence for a speed-accuracy trade-off.

### 4.3. Discussion

In line with the initial predictions, we found the space-time congruency effect in Experiment 3: There was a significant interaction between Time Position and Response Side in RTs as well as in accuracy.

## 5. Comparison Across Experiments

### 5.1. Reaction times

To statistically compare the results across the three experiments, we combined all data into a single model using the same approach described above for RT. All continuous variables were mean-centered. We created three dummy variables (one for each experiment), with two variables used in each analysis so that the remaining experiment always served as the baseline for pairwise comparisons. Because there was a substantial imbalance in the number of trials across experiments, we applied centered coding of the binary experiment factors this time.

The data were analyzed using a linear mixed model implemented in the R package *lme4*. The initial model included Time Position (continuous), Response Side (two levels), Experiment 1 (two levels), and Experiment 2 (two levels) as fixed factors, along with their three-way interaction (Time Position × Response Side × (Experiment 1 + Experiment 2)). Trial Number, Length, and Frequency were included as covariates. Random intercepts were specified for participants and items. (The full model with random slopes failed to converge.) To determine the best-fitting model, we performed a backward elimination using the *drop1* function, successively removing effects that did not improve model fit (*p* > .100). The main interaction of interest (Time Position × Response Side × (Experiment 1 + Experiment 2)) was retained regardless of significance.

Crucially, the model including the three-way interaction (Time Position × Response Side × (Experiment 1 + Experiment 2)) provided a significantly better fit than the model without it (*AIC* = –10330, *BIC* = –10243; *χ²*(6) = 49.16, *p* < .001). First, we used Experiment 3 as the baseline and compared the other two experiments to it. The interaction between Time Position and Response Side was significantly weaker in Experiment 2 than in Experiment 3 (*p* = .030), although the difference between Experiments 1 and 3 did not reach significance (*p* = .182). In the next step, we used Experiment 2 as the baseline and estimated the difference in the Time Position by Response Side interaction between Experiments 1 and 2, which also did not reach significance (*p* = .309). Detailed output of this analysis is provided in Table B7 (Appendix B).

### 5.2. Accuracy

To statistically compare the results across the three experiments, we combined all data into a single model using the same approach described in Section 5.1. All continuous variables were mean-centered. We created three dummy variables (one for each experiment), with two variables used in each analysis so that the remaining experiment always served as the baseline for pairwise comparisons. Because there was a substantial imbalance in the number of trials across experiments, we applied centered coding of the binary experiment factors this time.

We submitted the data to a generalized linear mixed-effects model (function *glmer*) with random intercepts for participants and items. (The full model with random slopes failed to converge.) The initial model included Time Position (continuous), Response Side (two levels), and Experiment (three levels) as fixed factors, along with their three-way interaction (Time Position × Response Side × (Experiment 1 + Experiment 2)). Trial Number, Length, and Frequency were included as covariates. Random intercepts were specified for participants and items. To determine the best-fitting model, we performed a backward elimination using the *drop1* function, successively removing effects that did not improve model fit (*p* > .100). The main interaction of interest (Time Position × Response Side × (Experiment 1 + Experiment 2)) was retained regardless of significance.

Crucially, the model including the three-way interaction (Time Position × Response Side × (Experiment 1 + Experiment 2)) provided a significantly better fit than the model without it (*AIC* = 11990, *BIC* = 12110; *χ²*(5) = 471.32, *p* < .001). First, we used Experiment 3 as the baseline and compared the other two experiments to it. The interaction between Time Position and Response Side was significantly weaker in Experiments 1 (*p* < .001) and 2 (*p* < .001) than in Experiment 3. In the next step, we used Experiment 2 as the baseline and estimated the difference in the Time Position by Response Side interaction between Experiments 1 and 2, which was not significant (*p* = .326). Detailed output of this analysis is provided in Table B8 (Appendix B).

### 5.3. Discussion

The findings from the RT and accuracy analyses indicate significant differences across the three experiments, likely due to the specific task demands (animacy-, space-, and time-relatedness). The significant pairwise comparisons of the key interaction (Time Position by Response Side) between experiments suggest that the relationship between Time Position and Response Side is modulated by the experimental context, with Experiment 3 consistently showing the strongest effect in accuracy as compared to the other two experiments and in RTs as compared to Experiment 2.

While the formal comparison across experiments did not reveal significant differences between Experiments 1 and 3 in RTs, this result should be interpreted with caution. As shown in Figure 1C, the overall RT pattern in Experiment 3 qualitatively differs from the other two experiments, with responses to future-related stimuli being numerically faster across all conditions – possibly due to an unknown task-related factor. As a result, the entire shape of the key interaction appears different compared to the other experiments. Therefore, in cases of inconsistency with the current cross-experiment analysis, we consider the results of the individual analyses reported in Sections 2–4 to be more reliable.

## 6. General discussion

The present study aimed to investigate the level of temporal demand sufficient to activate the attentional biases accompanying understanding of time-related words. To this end, we assessed the space-time congruency effect in tasks with varying levels of temporal demand while avoiding direct categorization of the word temporal dimension (past versus future categorization). In three consecutive experiments, we used the same set of past- and future-related words representing a temporal spectrum (ranging from distant past, through recent past and near future, to distant future). Participants were asked to categorize these words by using left and right response keys. Reaction times and accuracy were measured as dependent variables, while response side and words’ time position were included as independent variables. Importantly, each experiment employed a different word categorization task, thereby requiring distinct levels of temporal demand. In Experiment 1, words were categorized as living or non-living entities (word animacy task). Because the majority of studies avoiding temporal reference of the word failed to observe MTL activation, we expected to register no space-time congruency effect in this task. In Experiment 2, words were categorized as related to space or not (space-relatedness task). Based on the important role spatial language plays in the conceptualization of time, we expected that the space-time congruency effect should be observed in this task. In Experiment 3, words were categorized as being temporal or neutral (time-relatedness task). Since MTL activation has been demonstrated in tasks focusing on a broader temporal reference than the word temporal dimension, we expected that the space-time congruency effect would be observed in this task. To the best of our knowledge, this is the first study moving from the lower to higher levels of temporal demand while keeping the word temporal dimension (past versus future) irrelevant to the task. Furthermore, this study is one of the first MTL studies employing the categorization of temporal concepts based on their space- and time-relatedness.

Confirming our first hypothesis, Experiment 1 revealed no space-time congruency effect in the word animacy task. This aligns with previous research showing no MTL activation in tasks with lower temporal demand, such as determining the meaningfulness of sentences with temporal semantic aspect (Maienborn et al., 2015; Scheifele et al., 2018; Sell & Kaschak, 2011; Ulrich et al., 2012; Ulrich & Maienborn, 2010). Therefore, the findings from Experiment 1 generally support the idea that MTL activation is not functionally involved in conceptual processing with lower temporal demand (Maienborn et al., 2015; Santiago et al., 2011, 2012; Torralbo et al., 2006; Ulrich & Maienborn, 2010). While previous research has primarily focused on sentence-level processing, our study investigates whether the space-time congruency effect emerges in semantic processing at the level of single words. Categorizing words as living or non-living entities engages a fundamental semantic dimension that is largely activated automatically (Bonin et al., 2019). Given this, the word animacy task may serve as a baseline for future research on spatial-temporal mappings in tasks with lower temporal demand.

Moving from the lower to higher levels of temporal demand, we employed the space-relatedness task in Experiment 2. Confirming our second hypothesis, we observed the space-time congruency effect in this experiment. Specifically, participants responded faster to past-related words with the left key and to future-related words with the right key. To the best of our knowledge, this is the first study focusing on the spatial semantic aspects during processing of time-related words. Compared to the word animacy task, the space-relatedness task likely enhanced the activation of mental representations associated with temporal concepts, resulting in stronger spatial biases. Indeed, previous studies demonstrated a close link between temporal and spatial semantics, as well as the important role spatial language plays in the conceptualization of time (Akbuğa & Göksun, 2024; Lakoff, 1993; Lakoff & Johnson, 1999). Our findings provide further evidence of the bidirectional association between time and space (Anelli et al., 2016; Anelli & Frassinetti, 2019; Casadio et al., 2024; Walsh, 2003). The study demonstrates that in MTL research, the novel space-relatedness task might indeed evoke a greater temporal demand compared to a less semantically relevant aspect, such as word animacy.

Confirming our third hypothesis, Experiment 3 revealed the space-time congruency effect in the time-relatedness task. Notably, this effect was even more pronounced than in Experiment 2 since it was evident in both reaction times and accuracy: Participants reacted faster and more accurately to past-related words with the left key and to future-related words with the right key. While previous MTL studies primarily focused on reaction time data to assess attentional biases, our findings extend the methodological scope by highlighting accuracy as an additional parameter for investigating MTL activation (see Kühne et al., 2022, for other types of abstract concepts). Moreover, our findings are partially consistent with those of Malyshevskaya and colleagues (2024), who observed horizontal spatial biases for time units of different scales. In their study, verification questions regarding the specific class of the time unit were used. Drawing on a similar framework, we applied a broader reference to the concept of time *itself*, instead of past versus future categorization. To the best of our knowledge, this study provides one of the first demonstrations of the space-time congruency effect in a task requiring the highest level of temporal demand while keeping the word temporal dimension non-salient.

Overall, by manipulating the task demands, we demonstrated that MTL activation might occur when participants are asked to process time-related words based on their spatial and (general) temporal semantic aspects, but not on their relatedness to animacy. These findings are consistent with recent views on cognition, which suggest that mental representations are sensitive to context and environment (Barsalou, 1982, 1987; Fischer, 2012; Kaup et al., 2024; Kiefer & Harpaintner, 2020; Kiefer & Pulvermüller, 2012; Louwerse & Jeuniaux, 2008; Myachykov et al., 2014). Indeed, words possess multiple semantic aspects, and activation of a specific aspect depends on the contextual demands of the given task. In turn, the activated semantic aspect triggers the mental representations specific to it (Hoenig et al., 2008). For example, neurophysiological studies have demonstrated that processing the same word as a concrete object or as a living being leads to the activation of different regions of the brain (cf. Hargreaves et al., 2012; Van Dam et al., 2012). Modality-specific brain areas are activated depending on whether participants classify words performing sound or action judgments (Kuhnke et al., 2020). Given the bidirectional link between time and space processing and their shared neural bases (Cona et al., 2021), spatial cues in Experiment 2 might have activated time-related processes, which did not occur in Experiment 1. From this perspective, it is not unexpected that spatial-temporal mapping is revealed in some contextual tasks but not in others (Barsalou, 1987). Indeed, Barsalou (2020), emphasizing the context-sensitivity of mental representations, cautioned against expecting effects to generalize across all situations.

Moreover, our findings align with previous research suggesting that context affects the *degree* to which attentional and sensorimotor effects are activated, since we observed that the level of temporal demand modulated the space-time congruency effect. Indeed, stronger involvement of mental representations and therefore, stronger attentional and sensorimotor effects are often associated with tasks requiring higher conceptual demand (Goh et al., 2016; Ibáñez et al., 2022; Myachykov et al., 2014; Pexman et al., 2019; Shaki & Fischer, 2023). It therefore raises a need for future MTL research not to ignore the role of intermediate levels of temporal demand in the modulation of the space-time congruency effect. Specifically, our findings challenge the adequacy of the binary distinction between time being “task-relevant” or “task-irrelevant,” as proposed by previous MTL research (Maienborn et al., 2015; von Sobbe et al., 2019). For example, our Experiment 2 demonstrated that even when the word temporal dimension is task-irrelevant, the involvement of spatial semantic processing can still result in MTL activation. Moreover, the task used in Experiment 3 would traditionally fall under the “time is task-irrelevant” category, as past and future dimensions were not directly relevant to task performance (von Sobbe et al., 2019). However, the time-relatedness task in our study represented an intermediate level of temporal demand, which was sufficient to activate the MTL.

Finally, the present study investigated the space-time congruency effect using stimuli representing a temporal spectrum (ranging from distant past, through recent past and near future, to distant future). With MTL activation observed in Experiments 2 and 3, the results underscore the importance of distinguishing between categorical and continuous stimulus distributions in tasks avoiding processing of the word temporal dimension (e.g., past versus future). When experimental designs restrict participants to binary classifications, the results naturally reflect this dichotomy, framing temporal concepts in categorical terms. However, when the word temporal dimension is not directly relevant to task performance, temporal concepts are not artificially forced into categorical structure. Indeed, prior research has shown that MTL activation occurs in a continuous manner when the task avoids past versus future categorization (Beracci et al., 2022; Ding et al., 2015). Thus, future MTL research working with intermediate temporal demands should consider the continuous nature of time representation and avoid oversimplifying it through the use of artificial binary categories.

Regarding the pattern of results in Experiment 3, we found that in both hands, responses to future-related words were faster than to past-related words. While most studies on the space-time congruency effect predict, in an ideal-case scenario, a symmetric relationship between space and time (with faster and likely more accurate responses to past-related stimuli on the left side and to future-related stimuli on the right side), the asymmetrical pattern of results observed in Experiment 3 is not unusual (Grasso, Ziegler, Mirault, et al., 2022; Maienborn et al., 2015; Scheifele et al., 2018; Ulrich & Maienborn, 2010). Moreover, some MTL studies do not analyze asymmetries in result patterns and instead report results separately for response sides or time categories (Malyshevskaya, Fischer, et al., 2024; Miles et al., 2011; Torralbo et al., 2006; Weger & Pratt, 2008). When they do, asymmetries are often observed. For example, Weger and Pratt (2008) presented participants with left- vs. right-side targets following past- vs. future-related words. In Tables 2 and 3, they provide data aggregated by response side and time category. While they report only a significant interaction, without formal post-hoc comparisons for the entire Experiment 2A, one can observe a numerically larger difference between past- and future-related words for the right side than for the left side in Experiment 2A (Table 2). A similar tendency is evident in Experiment 2B for reaction times (Table 3), with an even reversed effect for past-related words – i.e., numerically faster responses to right-side cues after processing past-related words – seemingly contradicting the MTL hypothesis.

Asymmetric effects are therefore not uncommon in such studies. Various factors may contribute to these effects, including specific task demands, stimulus properties, or participant characteristics. We hypothesize that, in our case, the time-relatedness task used in Experiment 3 is the primary factor, as all other conditions were held constant across the three experiments. Specifically, Malyshevskaya et al. (2025) found in a recent study that younger participants tend to respond faster to future-related words than to past-related words, whereas the opposite pattern was observed in older participants. Since our sample primarily consisted of younger participants (*Mean* age = 24 years; *SD* = 6 years), this global effect – faster responses to future-related stimuli – may have overlapped with our effect of interest. This global effect “exerted pressure” on the right side of the expected interaction cross, making the negative slope for right-side responses even steeper, while the left-side positive slope moved closer to zero and even became slightly negative. Crucially, however, the overall relationship between the slopes remained unchanged (as shown in Figure 1C): Right-side responses to future-related stimuli were still faster than left-side responses.

### 6.1. Limitations and future directions

Our study has several potential limitations. First, because the number of past- vs. future-related words in any language is limited, we were forced to present each stimulus twice to reach a substantial number of trials. While we do not believe that the repetition of stimuli could substantially distort the pattern of results, it could nevertheless lead to a weaker MTL activation due to habituation (Roth et al., 2024). However, stimuli were repeated the same number of times in each of the three experiments, thus making the results comparable across experiments. Even if a habituation effect occurred during the double presentation of each stimulus, it would have been consistent across all three experiments and thus would not influence the validity of comparisons between them.

Second, we used different parts of speech in Experiment 1 for stimuli of interest and fillers. To compose the animacy judgment task, we had to include animal names. Unlike adverbs indicating time categories, animal names are nouns. Potentially, this difference could lead to more shallow processing of time-related stimuli as they could be distinguished from the task-relevant stimuli (names of animals) based on purely grammatical features. This limitation becomes more relevant since Experiments 2 and 3 included adverbs only. At the same time, no salient morphological markers distinguish nouns from adverbs in German. The only obvious difference between the two categories is the capitalization of nouns (e.g., “Fuchs” vs. “bald”, fox vs. soon). However, our participants could not rely on this visual feature to perform the task since we presented all stimuli in capital letters.

Third, our results could be influenced by markedness (cf. Nuerk et al., 2004): While all our stimuli of interest were associated with “no”-responses in Experiments 1 and 2 (Experiment 1: “Is it an animal?”; Experiment 2: “Is the word related to space?”), they were associated with “yes”-responses (“Is the word related to time?”) in Experiment 3. “No”-responses could be processed differently from “yes”-responses, e.g., by involving deeper cognitive processing as “no”-responses might be more effortful (cf. Deutsch et al., 2009). However, other studies on embodied semantics analyzed only “yes”-responses (e.g., Pecher et al., 2003, 2009) and still found expected effects. Furthermore, response times in Experiment 1 were shorter than in Experiment 3; it indicates that animacy judgments were easier for our participants than time judgments, despite the fact that “yes”-responses were required in Experiment 1 and “no”-responses in Experiment 3.

Finally, one could notice that some of the time-related stimuli have an internal reference to space. For example, the German word “vorgestern” contains the part “vor”, which also has the spatial meaning “in front of”. This illustrates once again the inseparable relationship between time and space, even at the historical level of language development. It might also have influenced the results of our Experiment 2, in which we asked participants to make spatial judgments. However, since we controlled for the space-relatedness of the two groups (past- vs. future-related stimuli) and found no significant differences between them, space-relatedness alone cannot explain why the two stimuli groups should have different left/right associations, which we found in Experiment 2.

## 7. Conclusion

To conclude, the present study provides new insights into the intermediate levels of temporal demand sufficient to activate the horizontal MTL. Specifically, we demonstrated the space-time congruency effect in tasks requiring the categorization of time-related words as related to space (Experiment 2) and time (Experiment 3), but not as living entities (Experiment 1). To our best knowledge, this is the first MTL study directly focusing on these semantic aspects in the processing of temporal concepts. Our findings support the notion that conceptual processing with higher levels of temporal demand leads to the stronger activation of mental representations resulting in stronger attentional biases. They are also consistent with those approaches to cognition that suggest the context-sensitive nature of mental representations.

## Data Accessibility Statement

The datasets generated for this study and processing scripts can be found in the Open Science Framework (OSF) at https://osf.io/zcwfs/ (doi:10.17605/OSF.IO/ZCWFS).

## Additional File

The additional file for this article can be found as follows:

10.5334/joc.448.s1 Appendices. Appendix A–D.
