# Supplementary material for: Does the Level of Temporal Demand Affect Activation of the Mental Timeline?
Source: J Cogn. 2025 May 28;8(1):37. doi: 10.5334/joc.448 (PMC12124278; doi:10.5334/joc.448)
Supplement: Appendices. — Appendix A–D. [file joc-8-1-448-s1.pdf]

Appendix A

Time-related stimuli used in all three experiments

German stimulus	English translation	Type	Time position	Length	Frequency (lgSUBTLEX)
DAMALS	back then	past	1.75	6	3.497
DAVOR	before	past	4.05	5	3.12
EHEMALS	formerly	past	2.125	7	1.748
EINST	once	past	1.5	5	2.724
EINSTMALS	in former days	past	1.575	9	0.845
FRÜHER	previously	past	1.825	6	3.583
GESTERN	yesterday	past	3.8	7	3.671
JÜNGST	recently	past	3.8	6	0.778
KÜRZLICH	lately	past	3.9	8	2.601
LETZTENS	the other day	past	3.5	8	2.217
NEULICH	newly	past	3.6	7	2.89
SEINERZEIT	at that time/then	past	1.9	10	1.146
UNLÄNGST	not long since	past	3.775	8	0.954
VERGANGEN	in the past	past	2.45	9	2.543
VORGESTERN	the day before yesterday	past	3.575	10	2
VORHER	before	past	3.875	6	3.403
VORHERIG	prior	past	3.75	8	1.556
VORHIN	a short while ago	past	4.05	6	3.087
VORJAHR	last year	past	2.6	7	0.903
ZUVOR	previously	past	3.9	5	3.153
ABSEHBAR	foreseeable	future	6.55	8	0.903
ANSCHLIEßEND	subsequently	future	5.95	12	1.908
ANSTEHEND	upcoming	future	6.575	9	1.342
BALD	soon	future	6.875	4	3.833
BEVORSTEHEND	upcoming	future	6.55	12	0.903
DANACH	after that	future	5.75	6	3.507
DEMNÄCHST	coming soon	future	6.675	9	2.233
FOLGEND	following	future	6.375	7	1.279
KOMMEND	forthcoming	future	6.875	7	1.519
KÜNFTIG	prospective	future	7.125	7	1.519
MORGEN	tomorrow	future	6.3	6	4.218
NACHHER	afterwards	future	6	7	2.913
NÄCHSTENS	coming next	future	6.525	9	0.699
NAHEND	approaching	future	6.475	6	2.021
SOFORT	immediately	future	5.175	6	3.974
SPÄTER	later	future	6.225	6	3.899
ÜBERMORGEN	the day after tomorrow	future	6.675	10	2.301
ZUKUNFT	future	future	8.4	7	3.519

ZUKÜNFTIG	in the future	future	7.9	9	1.279
ZUNÄCHST	at first	future	5.45	8	2.559

Note. Time position indicates word's past- vs. future-relatedness (ranging from 1, 'the most distant past', through 5, 'present', to 9, 'the most distant future'). Length indicates word's length in letters. Frequency indicates the log10 of word's frequency from Brysbaert et al. (2011).

Appendix B

Output tables with results

Table B1

Experiment 1 (word animacy task): output from the final model for RT

**random effects:**	**name**	**variance**	***SD***	
participant	intercept	0.0122	0.1106	
word	intercept	0.0006	0.0246	
residual		0.0290	0.1702	
**fixed effects:**	***b***	***SE***	***t*-value**	***p*-value**
(intercept)	6.3760	0.0115	555.1253	< .001
**time position**	**-0.0020**	**0.0023**	**-0.9026**	**.367**
**response side**	**-0.0045**	**0.0038**	**-1.2060**	**.228**
**time position*response side**	**-0.0030**	**0.0020**	**-1.5077**	**.132**
Covariates:				
trial number	-0.0003	< 0.0001	-13.1537	< .001
frequency	-0.0115	0.0041	-2.8207	.005

Note. Marginal R^2 = .019, conditional R^2 = .320.

Table B2

Experiment 1 (word animacy task): output from the model for accuracy

**random effects:**	**name**	**variance**	***SD***	
participant	intercept	1.3916	1.1797	
word	intercept	0.0371	0.1926	
**fixed effects:**	***Odds Ratios***	***SE***	***z*-value**	***p*-value**
(intercept)	70.9006	10.8691	27.7968	< .001
**time position**	**1.0579**	**0.0450**	**1.3222**	**.186**
**response side**	**0.9348**	**0.1432**	**-0.4403**	**.660**
**time position*response side**	**0.9117**	**0.0715**	**-1.1797**	**.238**
Covariates:				
trial number	1.0018	0.0008	2.1824	0.029
frequency	0.7039	0.0552	-4.4798	< .001

Note. Marginal R^2 = .038, conditional R^2 = .329.

Table B3

Experiment 2 (space-relatedness task): output from the final model for RT

random effects:	name	variance	*SD*	
participant	intercept	0.0130	0.1143	
word	intercept	0.0030	0.0551	
residual		0.0347	0.1862	
**fixed effects:**	***b***	***SE***	***t*-value**	***p*-value**
(intercept)	6.6481	0.0144	463.0586	< .001
**time position**	**0.0027**	**0.0047**	**0.5850**	**.559**
**response side**	**-0.0076**	**0.0046**	**-1.6574**	**.097**
**time position*response side**	**-0.0062**	**0.0023**	**-2.7039**	**.007**
		Covariates:		
trial number	-0.0003	< 0.0001	-10.3727	< .001

Note. Marginal R^2 = .012, conditional R^2 = .325.

Table B4

Experiment 2 (space-relatedness task): output from the model for accuracy

random effects:	name	variance	*SD*	
participant	intercept	0.5555	0.7453	
word	intercept	1.7535	1.3242	
**fixed effects:**	***Odds Ratios***	***SE***	***z*-value**	***p*-value**
(intercept)	8.9772	2.0326	9.6931	< .001
**time position**	**0.8731**	**0.0977**	**-1.2127**	**.225**
**response side**	**1.0260**	**0.0748**	**0.3519**	**.725**
**time position*response side**	**0.9841**	**0.0427**	**-0.3697**	**.712**
		Covariates:		
trial number	0.9997	0.9997	-0.7720	.440

Note. Marginal R^2 = .012, conditional R^2 = .420.

Table B5

Experiment 3 (time-relatedness task): output from the final model for RT

random effects:	name	variance	*SD*
participant	intercept	0.0087	0.0933
word		0.0044	0.0661

residual		0.0328	0.1810	
**fixed effects:**	***b***	***SE***	***t*-value**	***p*-value**
(intercept)	6.5490	0.0139	472.4755	< .001
**time position**	**-0.0090**	**0.0056**	**-1.6101**	**.107**
**response side**	**0.0002**	**0.0043**	**0.0522**	**.958**
**time position*response side**	**-0.0054**	**0.0024**	**-2.2983**	**.022**
		Covariates:		
trial number	-0.0003	< 0.0001	-5.9386	< .001
frequency	-0.0560	0.0101	-5.5618	< .001

Note. Marginal R^2 = .078, condition R^2 = .341.

Table B6

Experiment 3 (time-relatedness task): output from the model for accuracy

**random effects:**	**name**	**variance**	***SD***	
participant	intercept	0.3429	0.5855	
word	intercept	1.6385	1.2801	
**fixed effects:**	***Odds Ratios***	***SE***	***z*-value**	***p*-value**
(intercept)	13.5101	2.9278	12.0132	< .001
**time position**	**1.1103**	**0.1211**	**0.9596**	**.337**
**response side**	**0.4620**	**0.0366**	**-9.7494**	**< .001**
**time position*response side**	**1.8790**	**0.0776**	**15.2637**	**< .001**
		Covariates:		
trial number	0.9973	0.0008	-3.3196	.001
frequency	1.8653	0.3683	3.1568	.002

Note. Marginal R^2 = .160, conditional R^2 = .476.

Table B7

Comparison across experiments: output from the final model for RT

**random effects:**	**name**	**variance**	***SD***	
participant	intercept	0.0113	0.1061	
word		0.0013	0.0366	
residual		0.0331	0.1819	
**fixed effects:**	***b***	***SE***	***t*-value**	***p*-value**
(intercept)	6.5063	0.0080	810.3842	**< .001**
**time position**	**-0.0026**	**0.0028**	**-0.9422**	**.346**
**response side**	**-0.0056**	**0.0026**	**-2.1926**	**.028**
**time position*response side**	**-0.0027**	**0.0013**	**-2.0496**	**.040**

**experiment 1**	**-0.1565**	**0.0147**	**-10.6093**	**< .001**
**experiment 2**	**0.1215**	**0.0149**	**8.1470**	**< .001**
**response side*experiment 1**	**-0.0004**	**0.0065**	**-0.0545**	**.957**
**response side*experiment 2**	**-0.0045**	**0.0068**	**-0.6614**	**.508**
**time position*experiment 1**	**0.0051**	**0.0019**	**2.7355**	**.006**
**time position*experiment 2**	**0.0123**	**0.0019**	**6.3845**	**< .001**
**(time position*response side)*experiment 1**	**-0.0046**	**0.0035**	**-1.3339**	**.182**
**(time position*response side)*experiment 2**	**-0.0078**	**0.0036**	**-2.1752**	**.030**
		Covariates:		
trial number	-0.0003	0.0000	-17.0645	< .001
frequency	-0.0159	0.0050	-3.1749	.002

Note. Marginal R^2 = .255, condition R^2 = .460. In this model, Experiment 3 served as the baseline; that is, all comparisons for Experiments 1 and 2 were made with respect to it. In the follow-up model, we used Experiment 2 as the baseline; in that case, the term ((Time Position × Response Side) × Experiment 1) was compared to the analogous term from Experiment 2, which yielded no significant difference (b = 0.0031, SE = 0.0031, t = 1.0166, p = .309).

Table B8

Comparison across experiments: output from the final model for accuracy

**random effects:**	**name**	**variance**	***SD***	
participant	intercept	0.4466	0.6683	
word		0.7600	0.8718	
**fixed effects:**	***Odds Ratios***	***SE***	***z*-value**	***p*-value**
(intercept)	20.1408	2.7372	22.0946	< .001
**time position**	**1.0577**	**0.0732**	**0.8101**	**.418**
**response side**	**0.8206**	**0.0541**	**-3.0021**	**.003**
**time position*response side**	**1.1803**	**0.0412**	**4.7524**	**< .001**
**experiment 1**	**6.8378**	**0.9230**	**14.2428**	**< .001**
**experiment 2**	**0.5602**	**0.0607**	**-5.3435**	**< .001**
**response side*experiment 1**	**2.0058**	**0.3536**	**3.9483**	**< .001**
**response side*experiment 2**	**2.2522**	**0.2540**	**7.1995**	**< .001**
**time position*experiment 1**	**0.9045**	**0.0460**	**-1.9762**	**.048**
**time position*experiment 2**	**0.7378**	**0.0231**	**-9.7032**	**< .001**
**(time position*response side)*experiment 1**	**0.4176**	**0.0392**	**-9.3145**	**< .001**
**(time position*response**	**0.4549**	**0.0278**	**-12.9088**	**< .001**

**side)*experiment 2**				
Covariates:				
length	1.1908	0.0872	2.3849	.017

Note. Marginal R^2 = .269, condition R^2 = .465. In this model, Experiment 3 served as the baseline; that is, all comparisons for Experiments 1 and 2 were made with respect to it. In the follow-up model, we used Experiment 2 as the baseline; in that case, the term ((Time Position × Response Side) × Experiment 1) was compared to the analogous term from Experiment 2, which yielded no significant difference (*Odds Ratios* = 0.9178, *SE* = 0.0801, *z* = -0.9821, *p* = .326).

Appendix C

Descriptive statistics by condition (actual data, i.e., data without covariates)

Table C1

Descriptive statistics for reaction time data by Time Category and Response Side in all three experiments

Time Category	Past		Future	
Response Side	left	right	left	right
Experiment 1	601 (137)	601 (136)	602 (136)	597 (136)
Experiment 2	777 (185)	782 (184)	795 (191)	783 (183)
Experiment 3	715 (165)	711 (166)	711 (166)	702 (169)

Note. Values in cells represent *Mean* reaction times in ms. Values in parentheses represent *SD*s.

Table C2

Mean accuracy data by Time Category and Response Side in all three experiments

Time Category	Past		Future	
Response Side	left	right	left	right
Experiment 1	97%	98%	98%	98%
Experiment 2	84%	85%	83%	83%
Experiment 3	92%	78%	88%	88%

Appendix D

Back-transformed Accuracy results

Figure D1

Back-transformed predicted accuracy as a function of Response Side and Time Position in three experiments

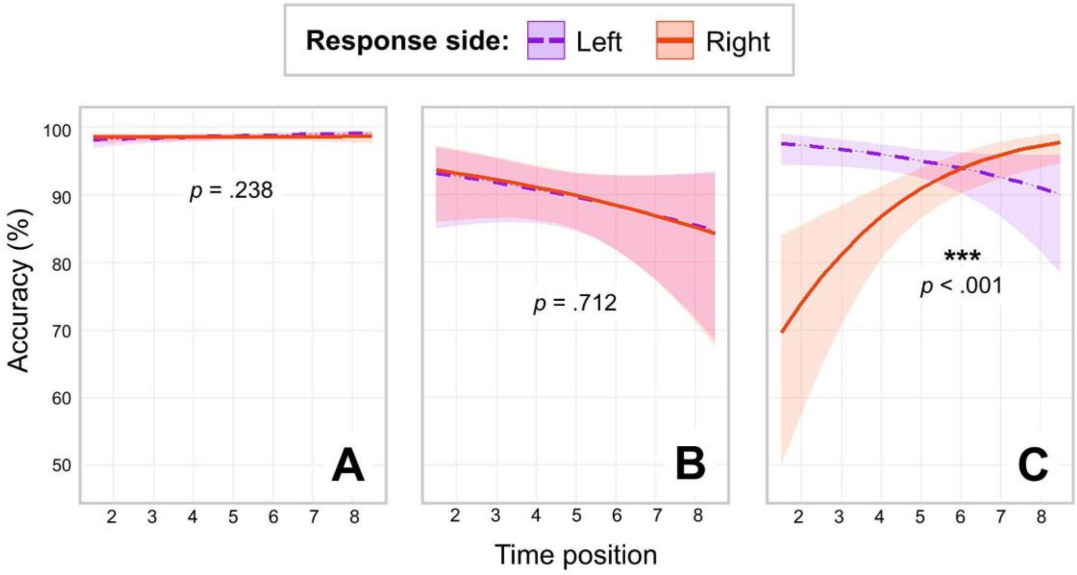

Note. Panel A: Results of Experiment 1 (word animacy task). Panel B: Results of Experiment 2 (space-relatedness task). Panel C: Results of Experiment 3 (time-relatedness task).
